# Immune-related adverse events in patients with low baseline serum IL-6 treated with durvalumab plus tremelimumab for hepatocellular carcinoma: a case series

**DOI:** 10.1007/s12328-025-02176-1

**Published:** 2025-07-09

**Authors:** Tsubasa Nobusawa, Shun Kaneko, Miyako Murakawa, Jun Tsuchiya, Masato Miyoshi, Fukiko Kawai-Kitahata, Mina Nakagawa, Sei Kakinuma, Yasuhiro Asahina, Ryuichi Okamoto

**Affiliations:** 1 Department of Gastroenterology and Hepatology, Institute of Science Tokyo, 1-5-45 Yushima, Bunkyo-ku, Tokyo, 1138519 Japan; 2 Center for Healthcare Education, Institute of Science Tokyo, Tokyo, Japan; 3 Department of Clinical and Diagnostic Laboratory Science, Institute of Science Tokyo, Tokyo, Japan

**Keywords:** Durvalumab plus tremelimumab (DT), Interleukin (IL)-6, Immune-related adverse events (irAE)

## Abstract

Systemic therapy for advanced hepatocellular carcinoma (HCC) has progressed significantly, yet the management of immune-related adverse events (irAEs) remains a challenge, particularly with anti-CTLA-4 and anti-PD-L1 antibodies. We present two cases of severe irAEs during durvalumab plus tremelimumab (DT) therapy, demonstrating successful management and favorable outcomes. In Case 1, a 76-year-old male with metabolic-associated steatotic liver disease-related HCC experienced disease progression after lenvatinib and subsequently initiated DT therapy. He developed Grade 3 immune-related myositis at week 5, which was successfully managed with prednisolone. A partial response (PR) was achieved and maintained, with a 55% tumor reduction at 8 months. In Case 2, a 73-year-old male with alcoholic cirrhosis and a 16-cm HCC received DT therapy. On day 19, he developed Grade 3 immune-related hepatitis and thyroiditis, which were controlled with prednisolone. After steroid tapering, durvalumab was successfully rechallenged, maintaining a 38% tumor reduction at 6 months with a sustained PR. In the case series, lower baseline serum interleukin (IL)-6 levels were associated with the development of Grade ≥ 3 irAEs. These findings suggest that IL-6 may serve as a predictive biomarker, aiding in risk assessment and the optimization of individualized treatment strategies prior to anti-CTLA-4 therapy.

**Supplementary Information:**

The online version contains supplementary material available at 10.1007/s12328-025-02176-1.

## Introduction

In the treatment of advanced hepatocellular carcinoma (HCC), combination therapies involving immune checkpoint inhibitors (ICIs), such as atezolizumab plus bevacizumab (AB) [1] and durvalumab plus tremelimumab (DT) [2], have become available. However, characterizing these therapies in real-world practice and making individualized treatment decisions remain significant challenges.

Since the incidence of immune-related adverse events (irAEs) is high with anti-CTLA-4 and anti-PD-L1 antibodies, and many cases require the use of high-dose steroids due to the severity of adverse events, appropriate management is essential [2]. We previously reported that, in various cancers excluding HCC, the median overall survival (OS) of patients who experienced any irAEs was significantly longer than that of patients without irAEs, and that the incidence of immune-related liver injury was significantly higher in patients treated with anti-CTLA-4 agents [3]. In clinical trials, the occurrence of irAEs was associated with improved OS in patients treated with DT, a finding generally consistent with observations for other ICIs [4]. However, data from actual clinical practice remains limited.

Therefore, we report two cases of HCC treated with DT therapy that, despite experiencing severe irAEs, showed favorable responses to treatment. In addition, we investigated predictive factors for the development of Grade ≥ 3 irAEs prior to DT therapy in patients treated at our hospital.

## Case reports

**Fig. 1:**
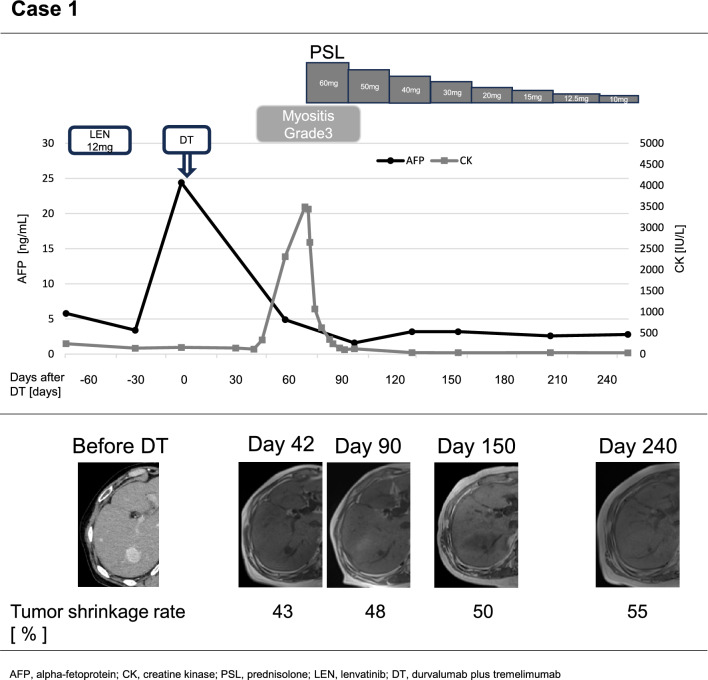
Clinical course of the patient developed Grade 3 immune-related myositis during durvalumab plus tremelimumab as 2nd line therapy. The changes in serum levels of alpha fetoprotein (AFP) and creatine kinase (CK) are shown. The tumor shrinkage rates are assessed mainly by non-contrast MRI due to renal dysfunction. *LEN* Lenvatinib, *DT* durvalumab plus tremelimumab, *PSL* prednisolone

**Fig. 2:**
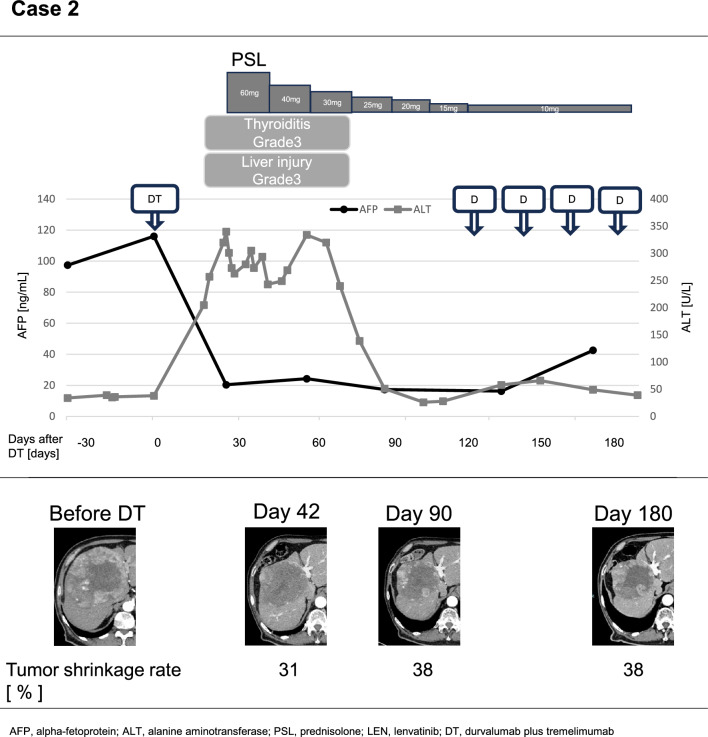
Clinical course of the patient developed Grade 3 immune-related liver injury and thyroiditis during durvalumab plus tremelimumab as 1st line therapy. The changes in serum levels of alpha fetoprotein (AFP) and alanine transaminase (ALT) are shown. The tumor shrinkage rates are assessed by contrast CT. *DT* durvalumab plus tremelimumab, *D* durvalumab, *PSL* prednisolone

(Case 1) A 76-year-old male underwent partial resection of segment 8 (S8) HCC against a background of metabolic-associated steatotic liver disease (MASLD). Thirteen months later, following multiple recurrences of HCC with up to five lesions measuring up to 2.4 cm in size, treatment with lenvatinib (LEN) was initiated. However, a CT scan one month later indicated progressive disease (PD) according to the Response Evaluation Criteria in Solid Tumors (RECIST). DT therapy was then administered as second-line systemic therapy. In the fifth week of DT therapy, the patient developed Grade 3 immune-related myositis. Prednisolone (PSL) at 1 mg/kg was initiated, resulting in good control of symptoms. The initial efficacy assessment showed a partial response (PR). Based on the patient’s own preference, systemic therapy was not resumed, and an observation approach was adopted. The patient expressed willingness to consider local therapy in the event of recurrence. Eight months after the initial single administration, a 55% tumor reduction was maintained without additional therapy (Fig. 1).

(Case 2) A 73-year-old male was diagnosed with esophagogastric junction cancer, and during further evaluation, a 16-cm HCC was also detected against a background of alcoholic liver cirrhosis. DT therapy was selected due to the risk of bleeding and the potential need for surgery for the esophagogastric junction cancer. On the 19th day of DT therapy, the patient developed Grade 3 immune-related liver injury and thyroiditis. PSL at 1 mg/kg was initiated, and after three months, the dose was gradually tapered to 10 mg before rechallenging with durvalumab. The initial efficacy assessment showed a PR. At the 6-month imaging follow-up, a 38% tumor reduction was maintained with continued durvalumab therapy (Fig. 2).

**Fig. 3:**
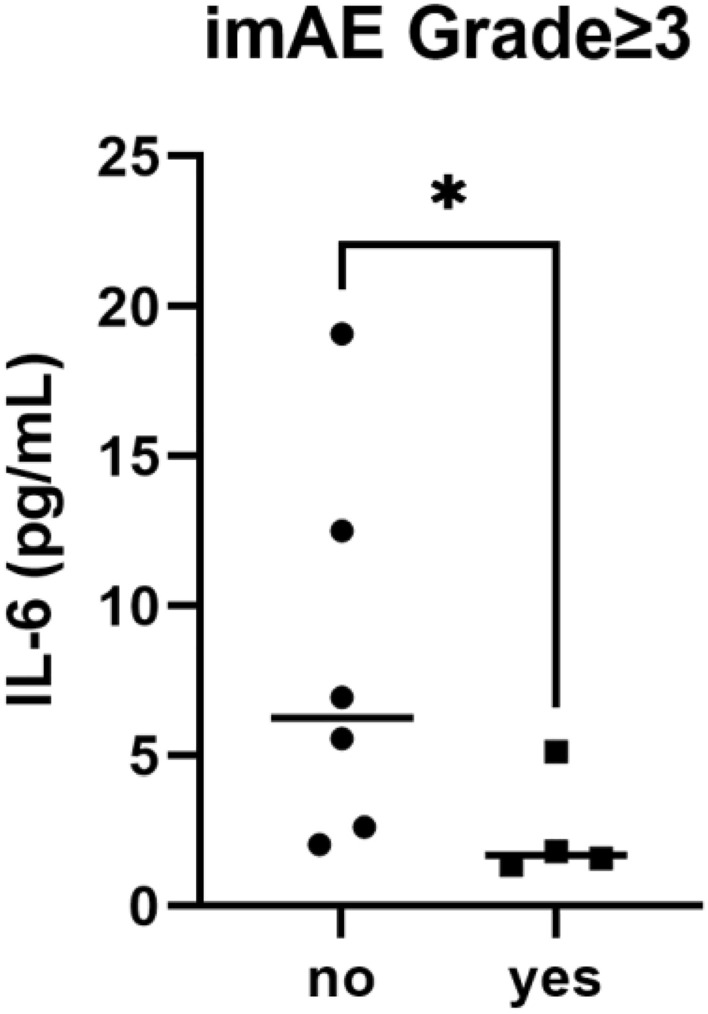
Baseline serum IL-6 levels in patients who experienced Grade ≥ 3 irAEs

A total of ten patients who received DT therapy were prospectively enrolled at the Institute of Science Tokyo Hospital between April 2023 and March 2024. Blood samples were collected from each patient during regular examinations, with the first sample obtained prior to the initiation of DT therapy. Serum cytokine levels were measured using the commercially available V-PLEX Proinflammatory Panel 1 Human Kit (Meso Scale Discovery, Rockville, MD), in accordance with the manufacturer's instructions. Among the ten patients who received DT therapy, four patients developed Grade ≥ 3 irAEs. As shown in Table 1, the Grade ≥ 3 irAEs included myositis, liver injury, thyroiditis, elevated pancreatic enzymes, and dermatitis. Each irAE was successfully managed with appropriate treatment. Disease control for HCC was achieved, as evidenced by radiological responses, including PR and stable disease (SD). Baseline serum IL-6 levels were significantly lower in patients who developed Grade ≥ 3 irAEs compared to those who did not (median: 1.705 pg/ml vs. 6.255 pg/ml; Mann–Whitney test, *p* = 0.0381; Fig. 3 and Supplementary Table 1).

**Table 1:** Summary of characteristics of patients who experienced Grade ≥ 3 irAE

No.	Age	Gender	irAE	Grade	Onset (days)	Tx for irAE	Anti-body at baseline	IL-6 (pg/mL)	Radiological response	ICI rechallenge
1 (Fig. 1)	76	Male	Myositis	3	45	PSL	Negative	1.36	PR	No
2 (Fig. 2)	73	Male	Liver injury Thyroiditis	3	17	PSL Levothyroxine	TPO antibody Thyroglobulin antibody	5.13	PR	Yes
3	80	Male	Elevated pancreatic enzymes	3	25	Nothing	Negative	1.82	SD	Yes
4	73	Male	Dermatitis	3	4	Steroid ointment	Acetylcholine receptor antibody	1.59	SD	No

*irAE* immune-related adverse events, *PSL* prednisolone, *TPO* thyroid peroxidase, *PR* partial response, *SD* stable disease

## Discussion

This valuable case series highlights the occurrence of Grade ≥ 3 irAEs in patients with low baseline serum IL‑6 who were treated with DT for HCC. Additionally, although the observation period and the number of cases were limited, we documented cases in which durvalumab could be rechallenged after successful management of irAEs, as well as a case where a single dose of DT alone achieved a sustained antitumor effect. The limited sample size posed challenges for conducting rigorous statistical analyses, which represents a limitation of the study. While the Mann–Whitney *U* test, supplemented by a Hodges-Lehmann estimation, provided statistically significant results, additional analyses using a previously reported IL-6 cut-off value (2.5 pg/mL) [5] showed that among patients with IL-6 < 2.5 pg/mL, three of four cases (75%) experienced Grade ≥ 3 irAEs, whereas only one of six cases (16.6%) was observed in the IL-6 ≥ 2.5 pg/mL group. However, Fisher’s exact test yielded a *p* value of 0.19, indicating no statistically significant difference, likely due to the small sample size and the resulting low statistical power. Nevertheless, this case series still allows for insightful and meaningful discussion.

In cancers other than HCC, particularly melanoma, research on biomarkers for irAEs is advancing, with a focus on cytokines [6]. Two studies have reported that lower baseline serum IL-6 levels were associated with the development of irAEs in melanoma patients treated with anti-CTLA-4 therapy. An analysis of baseline blood samples from 140 melanoma patients treated with anti-CTLA-4 therapy found that only low baseline IL-6 levels (< 2.5 ng/L) and female sex were correlated with an increased risk of Grade ≥ 3 irAEs after adjustment for follow-up time [5]. Similarly, lower levels of IL-6, IL-8, and sCD25 were associated with the development of anti-CTLA-4-induced colitis, as demonstrated using multiplex assays [7]. In patients with irAEs, a decreased frequency of regulatory T cells (Tregs) was observed, which was associated with more severe side effects and enhanced progression-free survival [8]. These findings suggest that patients with low baseline IL-6 levels may have a stable Treg population and a low-inflammatory, immunosuppressive environment. Anti-CTLA-4 therapy may disrupt Treg function, triggering immune dysregulation and increasing the risk of high-grade irAEs [9, 10]. Conversely, in HCC, high IL-6 levels have been reported as an unfavorable prognostic factor in patients treated with AB [11, 12]. More recently, IL-6 has also been reported as a prognostic factor in combination therapies such as radiotherapy and tislelizumab plus anlotinib [13]. Elevated CRP levels, which are closely linked to IL-6 activity, are also associated with poor prognosis in HCC, even during immunotherapy [14]. IL-6 is recognized as a major inducer of CRP and has been implicated in HCC progression [15]. IL-6 has been shown to promote tumor progression through the accumulation of myeloid-derived suppressor cells (MDSCs) [16]. Increased levels of MDSCs are associated with poor prognosis [17], and these cells can further enhance immunosuppressive pathways by inducing Tregs [18].

Taken together, these findings suggest that a low IL-6 environment may be characterized by reduced immunosuppressive cell activity, potentially allowing for enhanced antitumor responses during ICIs. However, such a state may also diminish the regulatory mechanisms that normally restrain immune overactivation, thereby increasing susceptibility to severe irAEs. Further research aiming to stratify patients based on this immunological “on–off” balance could facilitate safer and more effective implementation of immunotherapy in HCC.

From a clinical perspective, it is important to measure potential predictive and prognostic biomarkers such as IL-6 prior to administering DT. Comprehensive assessment of cardiac and pulmonary function, along with the patient's overall health status, is essential to evaluate their ability to tolerate potential severe irAEs.

In conclusion, low baseline IL-6 levels were significantly associated with the development of Grade ≥ 3 irAEs in HCC patients receiving DT therapy. However, appropriately managed severe irAEs may correlate with improved prognoses. Therefore, it is crucial to remain vigilant for irAEs both before and after initiating DT and to ensure early detection and prompt management.

## Supplementary Information

Below is the link to the electronic supplementary material.

Supplementary file1 (DOCX 21 KB)
