# Supplementary material for: Immune-related adverse events in patients with low baseline serum IL-6 treated with durvalumab plus tremelimumab for hepatocellular carcinoma: a case series
Source: Clin J Gastroenterol. 2025 Jul 9;18(5):899–904. doi: 10.1007/s12328-025-02176-1 (PMC12464045; doi:10.1007/s12328-025-02176-1)
Supplement: Supplementary file 1 — Supplementary file1 (DOCX 21 KB) [file 12328_2025_2176_MOESM1_ESM.docx]

**Supplementary Tables.**

**Table.1 Baseline clinical characteristics of patients treated with durvalumab plus tremelimumab (DT)**

|  | DT (N=10) | Non Grade≥3 irAE  (N=6) | Grade≥3 irAE  (N=4) | P value |
| --- | --- | --- | --- | --- |
| Age (years), median (range) | 74 (70-80) | 73.5 (70-78) | 74.5 (73-80) | 0.283 |
| Sex：Male (n, %) | 9 (90%) | 5 (83.3%) | 4 (100%) | 1.000 |
| BMI | 23.6 (19.2-28.4) | 22.3 (19.2-28.4) | 24.4 (22.5-27.9) | 0.088 |
| ECOG PS 0/1/2 | 7/3/0 | 4/2/0 | 3/1/0 | 1.000 |
| Etiology of CLD |  |  |  | 1.000 |
| HBV/HCV | 0 (0.0%) | 0 (0.0%) | 0 (0.0%) |  |
| Alcohol | 7 (70%) | 4 (66.7%) | 3 (75.0%) |  |
| Others | 3 (30%) | 2 (33.3%) | 1 (25.0%) |  |
| Child-Pugh Class A/B | 9/1 | 5/1 | 4/0 | 1.000 |
| ALBI score | -2.31 (-3.10 to -1.57) | -2.09 (-3.10 to -1.57) | -2.52 (-2.73 to -2.17) | 0.24 |
| mALBI grade 1/2a/2b/3 | 4/1/5/0 | 2/0/4/0 | 2/1/1/0 | 0.333 |
| AFP (ng/mL) | 20.5 (1.50-97.4) | 8.45 (1.50-45.1) | 13.5 (1.90-97.4) | 0.831 |
| NLR | 2.71 (1.46-4.40) | 2.70 (1.63-4.40) | 2.58 (1.46-3.77) | 0.521 |
| PLT (×10^4^/μl) | 17.3 (5.3-56.0) | 14.1 (5.3-56.0) | 10.1 (6.9-23.6) | 0.748 |
| Cirrhosis (n, %) | 7 (70%) | 5 (83.3%) | 2 (50%) | 0.5 |
| ALT (IU/L) | 31.6 (6.0-73.0) | 26.5 (16.0-43.0) | 38.5 (6.0-73.0) | 0.394 |
| CRP (mg/dL) | 1.06 (0.03-7.59) | 0.22 (0.03-7.59) | 0.24 (0.20-0.31) | 1.000 |
| **IL-6 (pg/mL)** | **5.87 (1.36-19.1)** | **6.26 (2.05-19.1)** | **1.71 (1.36-5.13)** | **0.033*** |
| BCLC stage |  |  |  | 0.2 |
| B (intermediate stage) | 7 (70%) | 3 (50%) | 4 (100%) |  |
| C (advanced stage) | 3 (30%) | 3 (50%) | 0 (0%) |  |
| Treatment line  1st/2nd/3rd/4th/5th/6th | 7/2/1/0/0/0 | 4/1/1/0/0/0 | 3/1/0/0/0/0 | 1.000 |
| 1^st^ vs beyond 2^nd^ | 7/3 | 4/2 | 3/1 | 1.000 |

DT, Tremelimumab plus Durvalumab; BMI, body mass index;

PS, performance status; CLD, chronic liver disease; HBV, hepatitis B virus; HCV, hepatitis C virus;

ALBI, albumin-bilirubin; mALBI, modified ALBI; AFP, Alpha fetoprotein;

WBC, white blood cells; NLR, neutrophil-to-lymphocyte ratio; PLT, platelet;

ALT, alanine aminotransferase; CRP, C-reactive protein; IL, interleukin;

BCLC, Barcelona Clinic Liver Cancer
